# Association of Initial Serum Total Calcium Concentration with Mortality in Critical Illness

**DOI:** 10.1155/2018/7648506

**Published:** 2018-06-26

**Authors:** Benji Wang, Yuqiang Gong, Binyu Ying, Bihuan Cheng

**Affiliations:** Department of Anesthesiology, Critical Care and Pain Medicine, The Second Affiliated Hospital and Yuying Children's Hospital of Wenzhou Medical University, Wenzhou, Zhejiang 325000, China

## Abstract

**Background:**

Several studies have suggested that serum ionized calcium (iCa) is associated with mortality in critical illness. However, evidence regarding the predictive significance of serum total calcium (tCa) in critical illness remains scarce. The aim of this study was to assess the association of tCa levels with mortality in critical illness.

**Methods:**

We employed the MIMIC-III v1.3 database. tCa was measured upon ICU admission and its relationship with mortality was determined using smooth curve fitting. The association between admission tCa levels and hospital mortality was determined using logistic regression.

**Results:**

Inclusion criteria were met by 44,886 critically ill patients. A U-shaped pattern was observed between tCa and hospital mortality. Similar trends were observed for hospital mortality when quintiles were used to group patients according to tCa. In multivariate analysis, adjusted for age and sex, the model indicated that admission tCa levels ⩽7.6mg/dl, 7.7-8.1mg/dl, and *⩾*9.0mg/dl were associated with an increase in mortality when compared to the reference level (8.6-9.0mg/dl). However, adjusted for more clinical characteristics, tCa was not associated with hospital mortality.

**Conclusions:**

The relationship between tCa and hospital mortality followed a ‘‘U” shaped curve. tCa had certain prognostic value in critically ill patients, but it had no independent association with hospital mortality.

## 1. Introduction

Electrolyte disturbances, including hypokalemia and hypocalcemia, affect many fundamental physiologic regulatory mechanisms [1, 2]. Particular aberrations in calcium metabolism can lead to serious cardiovascular complications and organ dysfunctions [3–5]. Derangement of ionized calcium (iCa) is widespread in patients with critical illness [6–8]. Several previous studies have studied the prognostic value of iCa in critical illness [7, 9, 10]. Most of these studies demonstrated that hypocalcemia was associated with increased mortality in critical illness [11–14], whereas there were few studies of hypercalcemia [15, 16].

Of total body calcium distribution, 98% is found in the skeleton, and the remaining 2% is found in the circulation. There are two main physicochemical states of serum total calcium (tCa). Approximately 50% of tCa is iCa, and the remainder is bound to albumin [17]. Thus, there is a close relationship between the tCa and iCa. Roland's study found that critically ill patients with lower tCa concentration have a high rate of lower iCa [18]. However, we do not know whether derangement in tCa was associated with mortality in critical illness.

Therefore, we extracted and analyzed the Multiparameter Intelligent Monitoring in Intensive Care Database III version 1.3 (MIMIC-III v1.3) to assess the impact of tCa on mortality in critical illness.

## 2. Methods

### 2.1. Data Source

MIMIC-III v1.3 comprises more than 40,000 intensive care unit (ICU) patients admitted to Beth Israel Deaconess Medical Center (Boston, MA, USA) from 2001 to 2012 [19]. To apply for access to the database, we completed the National Institutes of Health's web-based course and passed the Protecting Human Research Participants exam (NO. 6182750). We extracted clinical data, including patient demographics and laboratory test results. The establishment of this database was subject to approval by the institutional review boards of Massachusetts Institute of Technology and Beth Israel Deaconess Medical Center. To safeguard patient privacy, they were deidentified.

### 2.2. Population Selection Criteria

The MIMIC-III database recorded 58,976 ICU admissions. Adult patients (*⩾*18 years) were eligible. Patients were excluded if there was no tCa measured during ICU stay or if there were more than 10% data missing.

### 2.3. Data Extraction

Structured Query Language (SQL) with PostgreSQL (version 9.6) was used to extract data from MIMIC-III. The data extracted were clinical parameters, age, sex, temperature, respiratory rate, heart rate, blood pressure, blood oxygen saturation, comorbidities, laboratory parameters, and scoring systems. We extracted the following comorbidities: chronic heart failure, coronary artery disease, stroke, atrial fibrillation, chronic kidney diseases (CKD), acute kidney injury (AKI), pneumonia, liver disease, chronic obstructive pulmonary disease (COPD), malignancy, chronic pancreatitis, acute pancreatitis, and acute respiratory distress syndrome (ARDS). The following laboratory parameters were extracted: bicarbonate, creatinine, chloride, blood urea nitrogen (BUN), glucose, hematocrit, hemoglobin, platelet, sodium, and white blood cell (WBC). We also extracted sequential organ failure assessment (SOFA) and simplified acute physiology score II (SAPS II). Hospital mortality was the primary endpoint. tCa was measured on ICU admission. The baseline characteristic data were extracted within the first 24 h after patient ICU admission.

### 2.4. Statistical Analysis

Continuous variables are presented as mean ± standard deviation (SD) and compared using the analysis of variance or the Kruskal-Wallis test. Categorical variables were expressed as percentage and were compared using the chi-squared test.

The association between the tCa and hospital mortality was determined using a multivariate logistic regression and was expressed as the adjusted odds ratio with pertinent 95% confidence interval (CI). Two multivariate models were constructed on the basis of tCa according to quintiles derived with curve-fitting methods based on hospital mortality. The fourth quintile was considered as the control group. In model I, covariates were adjusted only for age and sex. In model II, covariates were adjusted for age, sex, oxygen saturation (SPO_2_), SOFA, systemic inflammatory response syndrome (SIRS), overall anxiety severity and impairment scale (OASIS), simplified acute physiology score II (SAPSII), systolic blood pressure (SBP), diastolic blood pressure (DBP), heart rate, respiratory rate, chronic heart failure, AKI, liver disease, acute pancreatitis, renal replacement therapy (RRT), anion gap, creatinine, chloride, glucose, and hemoglobin.

We performed stratification analyses to determine whether the effect of tCa differed across various subgroups. AKI, CKD, chronic heart failure, pneumonia, liver disease, acute pancreatitis, acute respiratory distress syndrome (ARDS), and RRT were also covered. All statistical analyses were performed using the EmpowerStats version 2.17.8 (http://www.empowerstats.com/cn/) and R software version 3.42. Two-tailed* P* value <.05 was considered to be statistically significant.

## 3. Results

### 3.1. Subject Characteristics

A total of 44,886 patients met inclusion criteria. Quintiles were used to group patients according to the tCa: 7,701 patients were in the ⩽7.6 mg/dl group, 8,382 patients were in the 7.7- 8.1 mg/dl group, 8,824 patients were in the 8.2- 8.5 mg/dl group, 10,219 patients were in the 8.6-9.0 mg/dl group, and 9,760 patients were in the *⩾*9.0 mg/dl group. Characteristics and hematologic laboratory data of the study patients according to admission tCa levels are displayed in Table 1.

Characteristics including age, sex, temperature, and SPO_2_ were relatively flat across each group. Blood pressure was lowest in severe hypocalcemia (⩽7.6 mg/dl), but heart rate and respiratory rate were highest. Patients with severe hypocalcemia were more likely to report a history of AKI, pneumonia, malignancy, chronic pancreatitis, and acute pancreatitis. Moreover, the proportion of comorbidities, such as stroke, CKD, and chronic obstructive pulmonary disease (COPD), were higher in those with severe hypercalcemia (*⩾*9.0 mg/dl). Participants with severe hypercalcemia also had higher levels of bicarbonate, creatinine, glucose, hematocrit, hemoglobin, platelets, sodium, and BUN, than did other groups. In addition, Elixhauser Comorbidity Index, SOFA, and SAPSII scores were significantly higher in severe hypocalcemia than in other groups.

### 3.2. tCa Levels and Hospital Mortality

**Figure 1:**
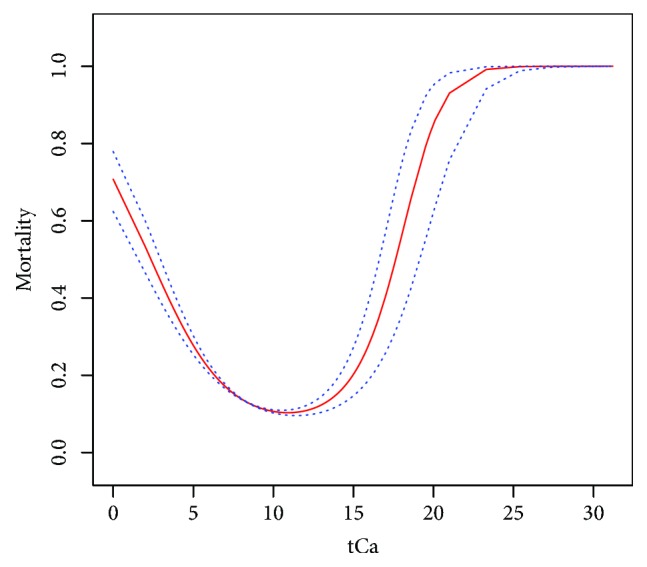
Relationship between initial tCa and logit-transformed mortality by using Lowess smoothing technique.

We divided initial tCa into variable categories.  Figure 1 shows the relationship between tCa and logit-transformed hospital mortality. The relationships were nonlinear. We observed a U-shaped relationship, suggesting that both hyper- and hypocalcemia were associated with equally increased risk of hospital mortality.

**Figure 2:**
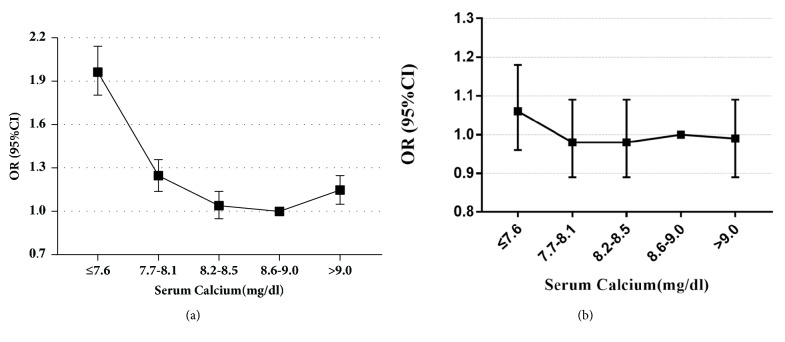
ORs (95% CIs) for hospital mortality across quintile groups of tCa ((a): model I, (b): model II).

In multivariate analysis, adjusted for clinical characteristics, including age and sex, the adjusted ORs (95% CIs) for admission tCa levels ⩽7.6mg/dl, 7.7-8.1mg/dl, and *⩾*9.0mg/dl compared to the reference level (8.6-9.0mg/dl) were 1.84 (1.69, 2.00), 1.23 (1.13, 1.35), and 1.12 (1.02, 1.22), respectively. These were linked to an increase in mortality. However, after adjustment for additional clinical characteristics including age, sex, SPO_2_, SOFA, SIRS, OASIS, SAPSII, SBP, DBP, heart rate, respiratory rate, chronic heart failure, AKI, liver disease, acute pancreatitis, renal replacement therapy, anion gap, creatinine, chloride, glucose, and hemoglobin, admission tCa levels were not independently associated with hospital mortality (Figure 2, Table 2).

### 3.3. Subgroup Analyses

The association between tCa and the risk of hospital mortality was similar for most strata (Table 3). Patients with AKI, CKD, chronic heart failure, pneumonia, liver disease, acute pancreatitis, and RRT had a significantly higher risk of hospital mortality for hypocalcemia, especially in severe hypocalcemia (⩽7.6 mg/dl). Patients with these diseases showed no difference in the risk of mortality for hypercalcemia. Neither hyper- nor hypocalcemia was linked with mortality in patients with ARDS. No matter what kind of the acid-base state, severe hypocalcemia increased the risk of hospital mortality. Hypercalcemia was associated with mortality only in acidosis. Although several tCa levels were related to the mortality in hypoalbuminemia, albumin had little effect on tCa in prognostic value of mortality.

## 4. Discussion

We demonstrated a U-shaped relationship between initial tCa and hospital mortality. Adjusted for clinical characteristics including age and sex, both hyper- and hypocalcemia were linked with increased hospital mortality in critically ill patients. When adjusted for several clinical characteristic, tCa was not associated with hospital mortality. Although many previous studies showed that iCa was associated with mortality in critical illness [20–23], the relationship between tCa and mortality had not been studied. To our knowledge, this was the first study to establish an association between initial tCa and mortality in critically ill patients.

Numerous risk factors appear to contribute to poor outcomes in critical illness. Although tCa was not an independent risk factor for mortality after adjustment for various clinical characteristics, it has a certain prognostic implication in critically ill patients. As observed in present study, a U-shaped relationship and a model that has been calibrated for age and gender indicated that tCa <8.2mg/l or >9.0mg/l were associated with hospital mortality. The greater the deviation from the normal value, the higher the death rate. In subgroup analysis, we showed that there was a correlation between tCa and hospital mortality for most critical illness disease categories. Thus, on ICU admission, the initial tCa level assessment could serve as a preliminary prognostic marker for mortality. However, we can not yet establish a predictive model of serum calcium for mortality. The precise prognostic value of tCa for mortality requires further investigation.

Calcium homeostasis is regulated by the vitamin D-parathyroid-calcium axis. Dysfunction of parathyroid hormone is highly prevalent in critical illness [24], and hypocalcemia is common in critically ill patients [25]. Potential mechanisms include proinflammatory cytokines impairing parathyroid hormone, hypersecretion of catecholamine, and redistribution of serum calcium [26, 27]. Nair's study [24] showed that vitamin D insufficiency or deficiency was prevalent among ICU patients. This prevalent vitamin D insufficiency may explain the high incidence of hypocalcemia. Severe hypercalcemia is associated with increase in mortality risk, attributable to either primary hyperparathyroidism or malignancy under most circumstances [28]. In addition, severe hypercalcemia is a risk factor for AKI in critically ill patients, and the occurrence of AKI would result in increased mortality [29, 30].

The major strength of this study is that we determine the association of tCa and hospital mortality for the first time. Mortality as primary outcome is clearly important in critically ill patients. In addition, the extracted data are from a large clinical database (MIMIC-III) and the number of patients enrolled is very large.

This study has a few limitations. First, the study has a single-center retrospective design. It is therefore subject to selection bias. Second, tCa was not an independent risk factor for mortality, but critically ill patients are heterogeneous. Whether increasing the specificity of the study will improve the prognostic value of tCa requires further investigation. Third, individual data are missing and outliers are present, possibly influencing the summary results. Finally, the follow-up length of mortality varied. Generally, 30-day mortality is the leading choice. We use hospital mortality, and this may result in too many instances of censored data.

## 5. Conclusions

In a large, heterogeneous group of critically ill patients, we found that the relationship between tCa and hospital mortality in critically ill patients followed a ‘‘U” shaped curve. tCa had a certain prognostic implication in critically ill patients but had no independent association with hospital mortality.

## Figures and Tables

**Table 1:** Characteristics of the study patients according to admission serum calcium levels.

Characteristics	Serum Calcium Levels (mg/dl)					P-value
	≤7.6	7.7- 8.1	8.2- 8.5	8.6- 9.0	≥9.0	
Clinical parameters, n (%)	7701	8382	8824	10219	9760	
Age, years	62.5±17.6	64.5±17.1	64.6±17.3	64.7±17.5	63.6±17.7	<0.001
Female, n (%)	3578(46.5%)	3551 (42.4%)	3804 (43.1%)	4517 (43.8%)	4642 (47.6%)	<0.001
SBP, mmHg	114.2±16.7	117.4±16.8	119.8±16.9	121.5±17.5	122.8±18.3	<0.001
DBP, mmHg	59.6±10.9	59.7±10.9	60.8±11.0	62.0±11.4	62.8±11.9	<0.001
Heart rate, beats/minute	90.5 ± 16.8	86.9 ± 16.2	84.7 ± 15.6	83.5 ± 15.6	83.8 ± 15.7	<0.001
Respiratory rate, beats/minute	19.6 ± 4.4	19.0 ± 4.1	18.9 ± 4.0	18.9 ± 4.0	18.9 ± 3.9	<0.001
Temperature, °C	36.9 ± 0.8	36.9 ± 0.6	36.8 ± 0.6	36.8 ± 0.6	36.7 ± 0.6	<0.001
SPO2, %	97.1 ± 3.6	97.2 ± 2.4	97.1 ± 2.4	97.0 ± 2.3	97.0 ± 2.4	<0.001
Comorbidities, n (%)						
Coronary artery disease	1400 (18.2)	1972 (23.5)	2222 (25.2)	2892 (28.0)	2442 (25.0)	<0.001
Chronic heart failure	951 (12.3)	1361 (16.2)	1497 (17.0)	2088 (20.2)	1881 (19.3)	<0.001
Atrial fibrillation	1802 (23.4)	2294 (27.4)	2406 (27.3)	2821 (27.3)	2450 (25.1)	<0.001
Stroke	381 (4.9)	584 (7.0)	809 (9.2)	1220 (11.8)	1304 (13.4)	<0.001
AKI	2627 (34.1)	2322 (27.7)	2115 (24.0)	2464 (23.9)	2528 (25.9)	<0.001
CKD	988 (12.8)	1192 (14.2)	1254 (14.2)	1712 (16.6)	1743 (17.9)	<0.001
Liver disease	687 (8.9)	646 (7.7)	567 (6.4)	583 (5.6)	677 (6.9)	<0.001
Pneumonia	2176 (28.3)	2042 (24.4)	2078 (23.5)	2327 (22.6)	2126 (21.8)	<0.001
COPD	155 (2.0)	206 (2.5)	220 (2.5)	324 (3.1)	340 (3.5)	<0.001
Malignancy	1659 (21.5)	1771 (21.1)	1719 (19.5)	1747 (16.9)	1697 (17.4)	<0.001
Chronic pancreatitis	98 (1.3)	91 (1.1)	93 (1.1)	89 (0.9)	73 (0.7)	0.005
Acute pancreatitis	312 (4.1)	210 (2.5)	183 (2.1)	164 (1.6)	163 (1.7)	<0.001
ARDS	141 (1.8)	138 (1.6)	157 (1.8)	173 (1.7)	179 (1.8)	0.812
Elixhauser Comorbidity Index	14.5 ± 13.2	12.7 ± 12.8	11.4 ± 12.4	10.7 ± 12.1	11.3 ± 12.2	<0.001
Laboratory parameters						
Bicarbonate, mmol/l	23.0 ± 4.5	24.7 ± 4.3	25.5 ± 4.2	26.2 ± 4.5	26.4 ± 4.8	<0.001
Creatinine, mEq/L	1.7 ± 2.0	1.5 ± 1.5	1.5 ± 1.5	1.6 ± 1.7	1.9 ± 2.2	<0.001
Chloride, mmol/l	109.4 ± 6.7	107.3 ± 6.0	106.1 ± 5.9	105.2 ± 5.9	105.2 ± 6.6	<0.001
Glucose, mg/dl	186.1 ± 123.4	171.9 ± 90.5	171.4 ± 94.7	173.9 ± 98.1	191.2 ± 128.7	<0.001
Hematocrit, %	34.0 ± 5.8	34.2 ± 5.7	34.9 ± 5.7	35.8 ± 5.7	37.1 ± 5.9	<0.001
Hemoglobin, g/dl	11.3 ± 2.1	11.4 ± 2.0	11.6 ± 2.0	12.0 ± 2.0	12.4 ± 2.1	<0.001
Platelet, 10^9^ /L	231.6 ± 133.7	244.7 ± 131.1	252.9 ± 127.6	263.3 ± 124.9	269.9 ± 131.1	<0.001
Sodium, mmol/L	140.3 ± 5.2	139.9 ± 4.7	139.9 ± 4.5	140.1 ± 4.5	140.6 ± 5.1	<0.001
BUN, mg/dl	30.8 ± 25.8	29.0 ± 23.1	28.1 ± 22.1	29.0 ± 22.8	31.9 ± 24.5	<0.001
WBC, 10^9^ /L	14.9 ± 10.8	14.0 ± 14.0	13.3 ± 9.6	13.2 ± 11.7	13.2 ± 11.4	<0.001
Scoring systems						
SOFA	5.3 ± 3.6	4.3 ± 3.1	3.9 ± 2.9	3.7 ± 2.8	4.1 ± 3.2	<0.001
SAPSII	39.2 ± 15.6	35.9 ± 14.5	34.2 ± 13.9	33.7 ± 13.8	35.0 ± 14.4	<0.001
Renal replacement therapy	403 (5.2)	353 (4.2)	335 (3.8)	476 (4.6)	709 (7.3)	<0.001

AKI: acute kidney injury; CKD: chronic kidney diseases; SBP: systolic blood pressure; DBP: diastolic blood pressure; COPD: chronic obstructive pulmonary disease; ARDS: acute respiratory distress syndrome; WBC: white blood cell; SOFA: Sequential Organ Failure Assessment; SAPSII: Simplified Acute Physiology Score II.

**Table 2:** Association between admission serum calcium levels and hospital mortality.

Serum Calcium	No. of patients	deaths	Non-adjusted		Model I^a^		Model II^b^	
(mg/dl)			OR (95%CI)	P value	OR (95%CI)	P value	OR (95%CI)	P value
Quintiles								
⩽7.6	7,701	1,443	1.84 (1.69, 2.00)	<0.001	1.97 (1.81, 2.15)	<0.001	1.06 (0.96, 1.18)	0.238
7.7-8.1	8,382	1,123	1.23 (1.13, 1.35)	<0.001	1.25 (1.14, 1.36)	<0.001	0.98 (0.89, 1.09)	0.768
8.2-8.5	8,824	1,018	1.04 (0.95, 1.14)	0.405	1.04 (0.95, 1.14)	0.371	0.98 (0.89, 1.09)	0.718
8.6-9.0	10,319	1,151	1.0(ref)		1.0(ref)		1.0(ref)	
>9.0	9,760	1,199	1.12 (1.02, 1.22)	0.013	1.15 (1.05, 1.25)	0.002	0.99 (0.89, 1.09)	0.781

OR: odds ratio; CI: confidence interval.

a: model I covariates were adjusted for age and sex.

b: model II covariates were adjusted for age, sex, spo2, SOFA, SIRS, OASIS, SAPSII, SBP, DBP, heart rate, respiratory rate, chronic heart failure, AKI, liver disease, acute pancreatitis, renal replacement therapy, anion gap, creatinine, chloride, glucose, and hemoglobin.

**Table 3:** Covariates were adjusted as in model I.

		Serum Calcium Levels (mg/dl)				
	N	≤7.6	7.7- 8.1	8.2- 8.5	8.6- 9.0	≥9
AKI						
No	32930	1.80 (1.60, 2.02)	1.10 (0.98, 1.25)	1.02 (0.91, 1.15)	1.0(ref)	1.16 (1.03, 1.30)
Yes	12056	1.74 (1.53, 1.98)	1.32 (1.15, 1.51)	1.07 (0.93, 1.23)	1.0(ref)	1.08 (0.94, 1.23)
CKD						
No	38097	2.03 (1.85, 2.22)	1.24 (1.12, 1.37)	1.03 (0.93, 1.14)	1.0(ref)	1.14 (1.03, 1.25)
Yes	6889	1.71 (1.38, 2.11)	1.30 (1.06, 1.61)	1.10 (0.89, 1.36)	1.0(ref)	1.19 (0.98, 1.44)
Chronic heart failure						
No	37208	1.91 (1.74, 2.10)	1.19 (1.08, 1.31)	1.01 (0.91, 1.11)	1.0(ref)	1.14 (1.04, 1.26)
Yes	7778	2.01 (1.62, 2.49)	1.47 (1.19, 1.81)	1.15 (0.93, 1.42)	1.0(ref)	1.17 (0.96, 1.43)
Pneumonia						
No	34237	2.18 (1.96, 2.42)	1.29 (1.15, 1.45)	1.01 (0.90, 1.13)	1.0(ref)	1.21 (1.08, 1.35)
Yes	10749	1.49 (1.29, 1.72)	1.14 (0.98, 1.32)	1.08 (0.93, 1.25)	1.0(ref)	1.07 (0.92, 1.24)
Liver disease						
No	41826	2.01 (1.83, 2.20)	1.23 (1.12, 1.36)	1.05 (0.95, 1.15)	1.0(ref)	1.12 (1.02, 1.22)
Yes	3160	1.37 (1.05, 1.79)	1.13 (0.86, 1.48)	0.92 (0.68, 1.23)	1.0(ref)	1.29 (0.99, 1.69)
Acute pancreatitis						
No	43954	1.95 (1.79, 2.13)	1.24 (1.13, 1.35)	1.04 (0.95, 1.14)	1.0(ref)	1.15 (1.05, 1.25)
Yes	1032	2.21 (1.27, 3.83)	1.54 (0.85, 2.81)	1.08 (0.57, 2.06)	1.0(ref)	1.24 (0.65, 2.37)
ARDS						
No	44198	1.99 (1.82, 2.17)	1.24 (1.14, 1.36)	1.04 (0.95, 1.14)	1.0(ref)	1.15 (1.05, 1.26)
Yes	788	1.33 (0.77, 2.32)	1.37 (0.79, 2.36)	1.08 (0.63, 1.88)	1.0(ref)	1.04 (0.61, 1.79)
RRT						
No	42710	1.99 (1.82, 2.17)	1.21 (1.11, 1.33)	1.04 (0.95, 1.14)	1.0(ref)	1.13 (1.03, 1.24)
Yes	2276	1.69 (1.21, 2.37)	1.86 (1.32, 2.61)	1.17 (0.81, 1.70)	1.0(ref)	1.10 (0.80, 1.50)
PH						
<7.35	9704	1.91 (1.65, 2.22)	1.21 (1.03, 1.42)	1.04 (0.88, 1.23)	1.0(ref)	1.29 (1.10, 1.50)
7.35-7.45	11850	1.67 (1.41, 1.98)	1.07 (0.90, 1.27)	1.11 (0.94, 1.30)	1.0(ref)	1.16 (0.99, 1.35)
≥7.45	5321	1.46 (1.15, 1.86)	0.91 (0.72, 1.15)	0.95 (0.77, 1.19)	1.0(ref)	0.90 (0.73, 1.11)
Albumin, g/dl						
1 - 2.8	5927	1.02 (0.84, 1.24)	0.76 (0.62, 0.94)	0.91 (0.72, 1.15)	1.0(ref)	1.16 (0.90, 1.49)
2.9 - 3.4	5693	0.89 (0.71, 1.11)	0.84 (0.68, 1.05)	0.89 (0.72, 1.11)	1.0(ref)	1.29 (1.03, 1.61)
3.5 - 6.3	6881	1.35 (0.98, 1.87)	1.02 (0.76, 1.37)	0.91 (0.71, 1.17)	1.0(ref)	1.11 (0.92, 1.35)

AKI: acute kidney injury; CKD: chronic heart failure; ARDS: acute respiratory distress syndrome; RRT: renal replacement therapy.

## Data Availability

The data used to support the findings of this study are available from the corresponding author upon request.
